# Soft Tissue Phantoms for Realistic Needle Insertion: A Comparative Study

**DOI:** 10.1007/s10439-015-1523-0

**Published:** 2015-12-14

**Authors:** Alexander Leibinger, Antonio E. Forte, Zhengchu Tan, Matthew J. Oldfield, Frank Beyrau, Daniele Dini, Ferdinando Rodriguez y Baena

**Affiliations:** 1 Department of Mechanical Engineering, Imperial College London, Exhibition Road, South Kensington, London, SW7 2AZ UK; 2 Lehrstuhl für Technische Thermodynamik, Otto-von-Guericke-Universität Magdeburg, Magdeburg, Germany

**Keywords:** Minimally invasive surgery, Brain, Tool-tissue interactions, Digital image correlation, Strain imaging, Soft tissue biomechanics, Gelatin

## Abstract

Phantoms are common substitutes for soft tissues in biomechanical research and are usually tuned to match tissue properties using standard testing protocols at small strains. However, the response due to complex tool-tissue interactions can differ depending on the phantom and no comprehensive comparative study has been published to date, which could aid researchers to select suitable materials. In this work, gelatin, a common phantom in literature, and a composite hydrogel developed at Imperial College, were matched for mechanical stiffness to porcine brain, and the interactions during needle insertions within them were analyzed. Specifically, we examined insertion forces for brain and the phantoms; we also measured displacements and strains within the phantoms *via* a laser-based image correlation technique in combination with fluorescent beads. It is shown that the insertion forces for gelatin and brain agree closely, but that the composite hydrogel better mimics the viscous nature of soft tissue. Both materials match different characteristics of brain, but neither of them is a perfect substitute. Thus, when selecting a phantom material, both the soft tissue properties and the complex tool-tissue interactions arising during tissue manipulation should be taken into consideration. These conclusions are presented in tabular form to aid future selection.

## Introduction

Working with real specimens of soft tissue for experimental studies in the biomechanical field presents a number of difficulties; in addition to ethical regulations and scarce availability, the data obtained is often unreliable or inconsistent due to complex tissue properties and testing protocols. Brain, especially, has properties that vary between *in vivo* and *in vitro* conditions^2^ and is highly sensitive to factors such as post-mortem time, sample preparation or mechanical history.^6^ Soft tissue specimens are also cumbersome to fix and constrain, leading to complex experimental designs^7^,^12^ and varying conditions between experiments. These problems have led to large deviations between experimental results in literature, which make comparisons across different studies difficult.^11^

For these reasons, soft tissues are commonly replaced by phantoms for experimental studies, as they are more controllable, easier to handle, and eliminate the problem of sample-specific variations. These advantages are exploited in fields ranging from surgical training to the modelling of interactions between soft tissue and tools, for instance in the context of percutaneous intervention where phantoms are used to validate models of needle insertions^3^,^15^ and to find and demonstrate new concepts for insertion methods with reduced tissue motion^20^ and for needle steering with enhanced accuracy.^10^

Phantom materials should mimic the relevant properties of soft tissue closely in order to translate findings to medical procedures. There is a large variety of phantom materials within the literature, with a broad range of material properties, depending on the relevant application and objectives. Materials are usually selected based on typical mechanical characterization protocols, such as compression, tensile or indentation tests^3^,^18^ or for best desired performance, e.g., to maximize the curvature of a steering needle.^25^

During needle insertions, large strains and cutting cause complex tool-tissue interactions, and phantoms should mimic soft tissue sufficiently for all domains of interest during the insertion. One of the most common soft tissue phantoms for modelling interactions, due to its organic nature and ease of handling, is gelatin.^5^,^22^,^24^ Gelatin phantoms are primarily characterized by their stiffness and are generally tuned by modifying the gelatin concentration, in order to match properties of soft tissue. However, gelatin has a near linear elastic behavior, with lower rate dependency compared to soft tissue, which is generally highly viscous.^14^ Conversely, a composite hydrogel (CH) developed at Imperial College London^4^ to simulate brain in compression, indentation, relaxation, shear and hysteresis, shows very good agreement with brain tissue for stiffness- and viscosity-related properties, which advocate its use as a better synthetic phantom compared to gelatin.

The goal of this work is to evaluate the performance of transparent gelatin and a modified, transparent version of the composite hydrogel (MCH), in the context of needle insertions. *In vitro* needle insertions are performed on these materials and compared to real soft tissue, porcine brain, which is known to behave similarly to human brain.^6^,^16^

The stiffness of the phantoms was first tuned to match brain using a standard indentation test. Fracture tests were conducted in order to characterize the behavior at failure of all phantoms (gelatin, CH, MCH) and real tissue. We then measured the required insertion forces for brain, MCH and gelatin; due to the lack of transparency of biological soft tissue, for the phantoms only, we examined internal displacements and strains close to the needle using a laser-based imaging technique, which allows a comprehensive measurement of the interactions between the inserted needle and the substrate.^19^ Based on these measurements during the needle insertion process with both materials, conclusions can be drawn on the suitability of either phantom as a realistic alternative to real soft tissue.

The following section provides a detailed description and characterization of the materials used and the experimental setup devised for this study. This is followed by the results of the insertion forces measured for the synthetic materials, which are compared to porcine brain samples. A closer comparison of resulting displacements and strains for gelatin and MCH, due to fracture, friction, and viscous effects, is then provided, followed by a discussion and conclusions, which address the different characteristics of the materials and how they apply to studies that may require a realistic tissue phantom.

## Materials

### Materials

The composite hydrogel was composed of Phytagel (PHY), polyvinyl alcohol (PVA, 146,000–186,000 molecular weight) and deionized water, all supplied by Sigma-Aldrich Co., USA. The bovine gelatin powder was provided by Sleaford Quality Foods Ltd., UK. All concentrations of the phantom materials in the following sections are expressed as a percentage by mass (wt%).

Two days post-mortem porcine brain samples (2 samples) were obtained from a local supplier.

### Sample Preparation

To replicate the same boundary conditions across experiments, all samples were filled into transparent acrylic boxes, with an open top, an inner cross section of 80 × 80 mm^2^ and a height of 50 mm.

The MCH was obtained by modifying the procedure for CH described in Forte *et al*.^4^ to create a transparent composite hydrogel that could be used for non-invasive, highly resolved optical measurement within the samples. Specifically, the freezing step was removed from the procedure to avoid gelation of the PVA. The resultant network is phase separated, with the PHY forming the continuous polymer network (dominant phase) and the PVA dissolved as the filler network (included phase).

The MCH was produced by separately dissolving PVA and PHY in deionized water for 1 h at 90 °C. The two solutions were then mixed together in a 1:1 weight ratio at 70 °C, under constant stirring for 30 min. Particular care was used to avoid evaporation during the process. The solution was seeded with fluorescent melamine resin beads to enable imaging (size 10 *μ*m, rhodamine B-marked, Sigma Aldrich Co.), and poured into the transparent boxes. The boxes were covered with cling film to limit evaporation and slowly cooled at room temperature for 7 h before testing.

Due to the modified procedure, the MCH exhibited different mechanical characteristics from the original CH, which had a coupled network structure due to the presence of hydrogen bonds. Therefore, the concentrations of PVA and PHY needed to be tuned in order to match the stiffness of brain tissue, as described in “Stiffness Matching” section. The result is a transparent, highly viscous gel, which can also be used for internal optical measurements.

Gelatin gels were produced by mixing deionized water and gelatin powder. Deionized water was heated to 90 °C, and gelatin powder was then added and stirred into the water for 10 min. The solution was seeded with the same particles as the MCH and left to cool at room temperature. Cling film was also used and samples were stored in a domestic refrigerator, kept at 14 °C for 12 h; samples were tested on the following day, after the gel had reached room temperature (22 ± 2 °C).

The micrometer-sized beads used to seed the phantoms had a density of 1.51 g/cm^3^, which is sufficiently close to the density of the phantoms, mainly consisting of water. This allowed the added particles to distribute evenly in the phantom before solidification. The concentration of the aqueous solution with the particles in the phantoms was approximately 0.04 vol%, resulting in a particle number density of 16 particles/mm^3^. This resulted in a seeding density suitable for the imaging process (see “Methods” section). Indentation tests showed that the particles did not influence the material behavior due to their small size, similar density, and low number density.

Porcine brain samples were collected from the butcher immediately after being removed from the dura, then stored in a physiological solution at 4 °C during transportation. Specimens were not frozen at any time during the procedure.

### Stiffness Matching

**Figure 1:**
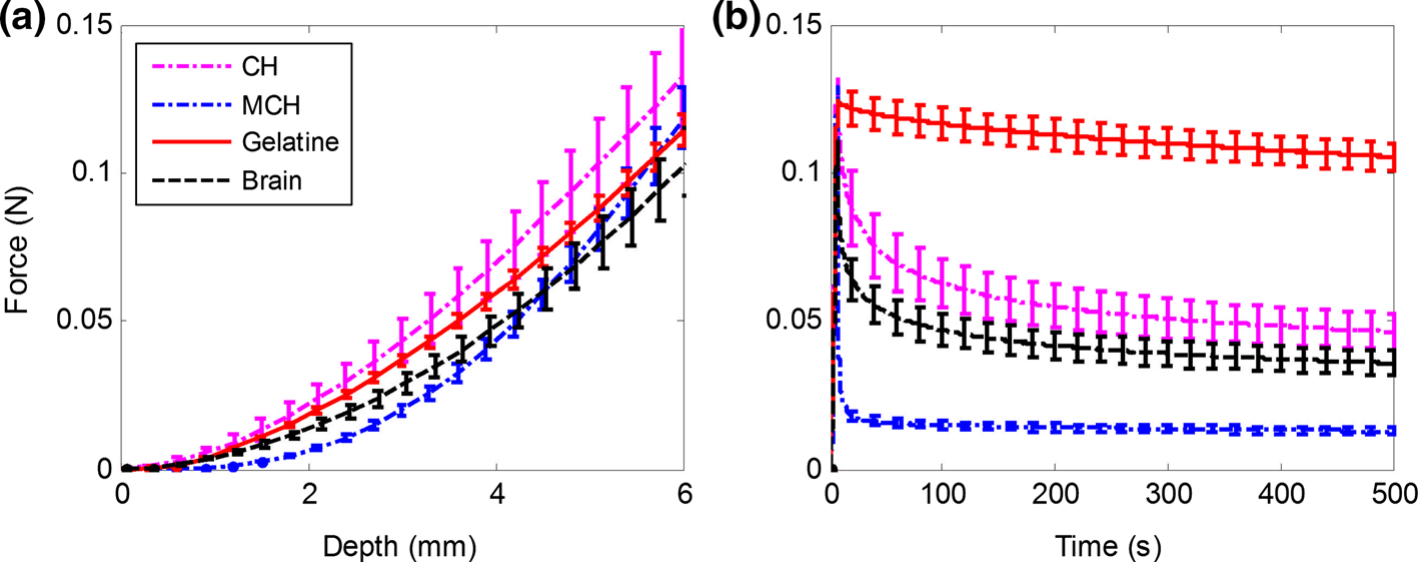
Compression and relaxation tests on porcine brain, gelatin, CH and MCH. The overlapping curves in the compression step (a) demonstrate very similar elastic properties for all phantom materials to porcine brain; however, gelatin exhibits a different relaxation behavior, characterized by a very small decay in the relaxation plot (b). For this reason, porcine brain tissue, CH and MCH are considered viscoelastic materials, while gelatin is considered nearly elastic.

The stiffness of the CH, MCH and gelatin was tuned to match the stiffness of the porcine brain tissue by varying the concentration of the powders dissolved in deionized water. An indentation test protocol, carried out with a mechanical testing system (Mach-1, Biomomentum Inc., CA) with a 150 g load cell (7.5 mg load resolution) and a spherical indenter (3.175 mm radius), was performed to compare samples that filled the same boxes as used for the needle insertions. Gefen *et al*.^7^ suggested that the indenter tip radius should be no more than 25% the thickness of the tested sample and they used an indentation depth equal to the diameter of the indenter. Therefore, the indentation depth was set to 6 mm. The test was performed at a displacement rate of 1 mm/s. After reaching the maximum depth, the indenter was kept in position for 500 s to record the relaxation behavior of each material. Five repetitions were carried out for each material. Figure 1a shows the indentation curves for the brain samples and the tuned phantom materials of gelatin (3.4%), MCH (3% PVA + 0.75% PHY), CH (PVA 5% + PHY 0.59%), respectively. The indentation curves for porcine brain tissue and the phantoms show very good agreement and were considered sufficiently close to each other to assume similar stiffness properties. Because of their viscous nature, the relaxation behavior of the composite hydrogels (CH and MCH) is closer to that of brain tissue, whereas gelatin, as expected, behaved as a nearly elastic material (Fig. 1b). The MCH relaxes more quickly than the original CH due to its weakly bonded network (“Sample Preparation” section). Knowledge of the difference in viscous properties of the phantom materials will allow us to draw certain conclusions from the measured interactions with the inserted needle.

### Fracture Behavior

Fracture tests were performed on brain and the phantom materials. Cylindrical samples (diameter 14 ± 1 mm, height 9 ± 1 mm) were tested in compression, using the same mechanical testing system and with the same displacement rate as for the needle insertions. Each sample was compressed between two plates until failure was detected. Silicon oil was applied at the interface between the sample and the compression plates in order to minimize friction effects.^5^ Uniform expansion of the sample in the radial direction was monitored. True strains and stresses were computed from recorded forces and displacements.

**Figure 2:**
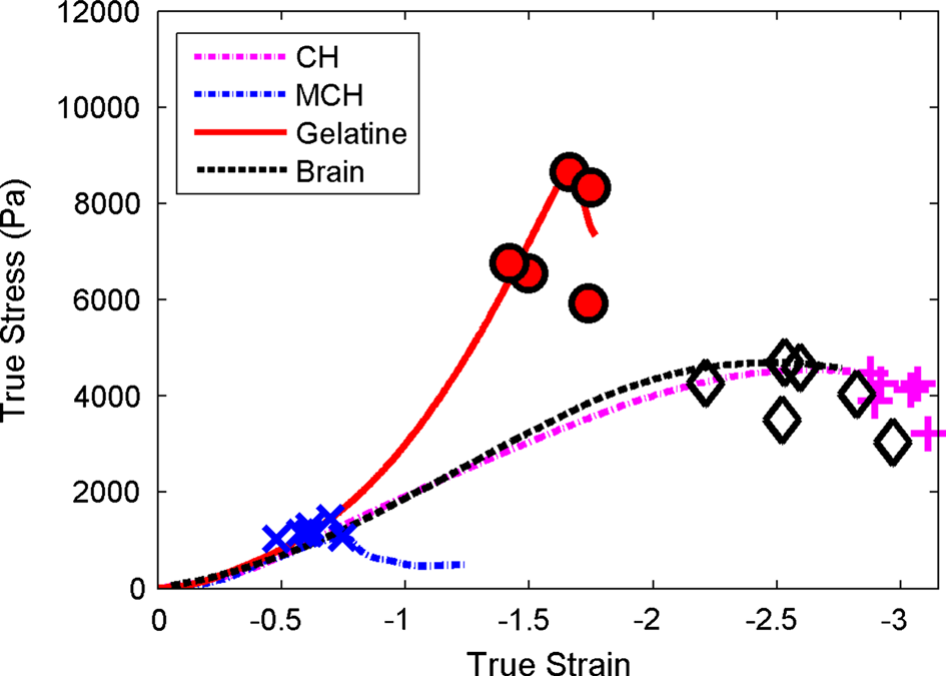
Compression to failure test results for brain tissue and the phantom materials. Despite having the same response at small strain (tuned *via* indentation tests), the three phantom materials show a diverging behavior when approaching failure. CH shows good agreement to brain at all stages.

In Fig. 2, the true stresses and strains are shown for the point of material failure during compression testing, for each material tested. Fracture becomes typically visible by a sudden drop in the stress–strain curve. Typically, the brain and the CH samples did not break during testing and the values presented in Fig. 2 represent the point when the two compression plates came almost into contact, compressing to 95% of the sample’s height.

All four materials show a similar response at the small strains for which they were matched, but a diverging behavior at the point of failure. The brain tissue and CH show true stresses at failure in the same range of approximately 3–4.5 kPa. The CH shows a ductile and stretchable behavior, which is very similar to brain tissue. MCH shows the lowest final stresses and strains, as it fails early on in the tests. Compared to brain, gelatin shows more brittle fracture behavior, with high stresses and low strains, whereas the behavior of brain and CH can be described as ductile, with larger deformations.

## Methods

The needle insertions were performed using the same mechanical testing system. A straight needle, with a conical tip with a 40° included tip angle and an outer diameter (OD) of 4 mm, was manufactured out of a rigid rapid prototyped material (Elastic Modulus 1.7–2.1 GPa, Endur, Stratasys Ltd., USA), connected directly to the load cell.

**Figure 3:**
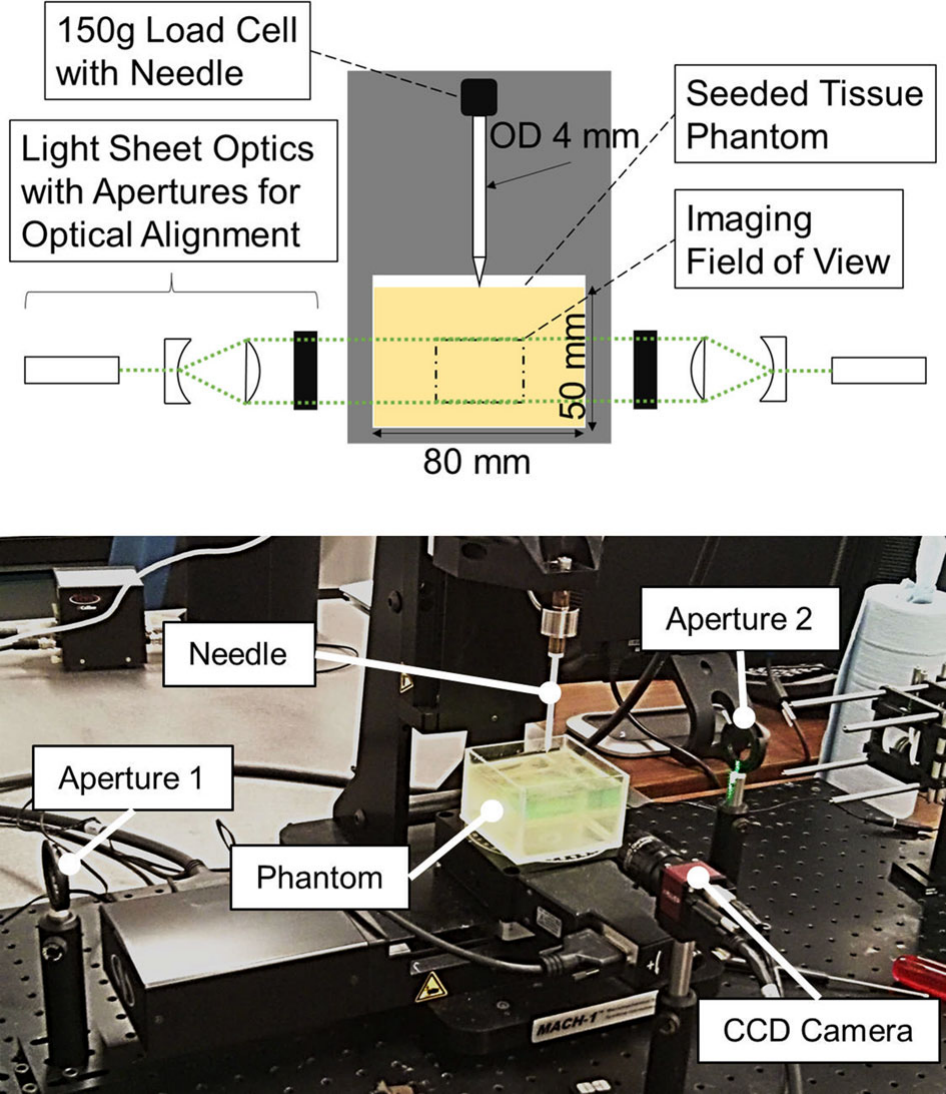
Schematic of the experimental setup (top) and picture during testing (bottom) showing the needle and the soft tissue phantom. Forces are recorded by the load cell and the imaging optics are arranged around the mechanical testing system.

The needle was inserted from the top and exited through a hole in the bottom of the box (Fig. 3). Eight insertions were performed for each phantom material, and four insertions were performed on porcine brain. In order to produce equivalent boundary conditions between materials, the brains were placed in (and fully filled) the same boxes as those used with the phantoms. The sample height was 43 ± 2 mm for all insertions. The needle was inserted with a speed of 1 mm/s, travelled through the sample for an additional 20 mm after exiting the box, and was held in position for 30 s before being retracted. This insertion speed is within a range commonly used in brain.^13^ After each insertion, the needle was directly inserted a second time in the pre-existing crack (reinsertion) to evaluate the contribution of cutting to the measured interactions.

Throughout the insertion and retraction, full field displacements and strains around the needle inside the transparent phantoms were measured using a laser-based image correlation technique,^9^ which has been used for the detailed investigation of tool-tissue interactions.^17^,^20^ The light emitted by the embedded fluorescent microbeads in the phantoms was captured by a charge-coupled device (CCD) camera (Guppy Pro, Allied Vision Technologies GmbH, Germany) at 7.5 frames per second, in a measurement plane aligned to the needle axis. This frame rate was used to ensure that enough particles stayed within the light sheet between captured frames for successful image correlation. The field of view ended 15 mm above the bottom of the box, had a size of approximately 20 × 15 mm^2^ and was calibrated for each experiment. Laser optics and two 532 nm diode laser sources (4.5 mW collimated DPSS laser, Thorlabs Inc., USA) on opposite sides of the needle created two coinciding light sheets, with a thickness of 4 mm, which ensured that both sides of the needle were illuminated at all times.

**Figure 4:**
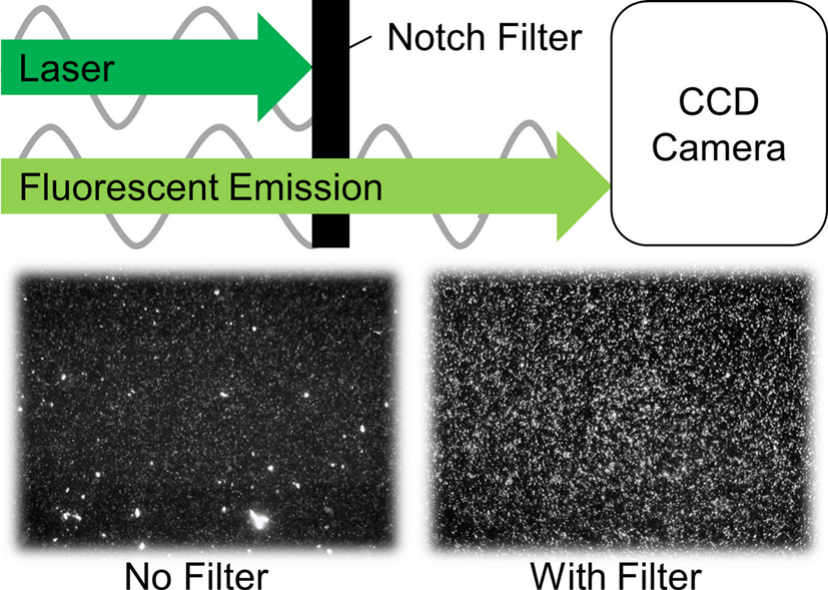
The wavelength filter blocks the laser at its specific frequency of 532 nm, while transmitting the emitted particle light at a shifted wavelength, with a peak at 584 nm. Recorded frames at the bottom show how scatter objects that are not particles (left) “disappear” by using the filter (right).

For improved imaging, experiments were carried out in a dark room, and fluorescent rhodamine B-marked particles were used, with an excitation wavelength around 540 nm and an emission peak at 584 nm. Using a notch filter placed in front of the camera optics, the laser light was blocked out spectrally and only the fluorescent particle emission was captured by the camera (Fig. 4). With the filter, PVA pieces “floating” in the MCH are no longer visible in the recorded images, leaving only the particles in clear view (see Fig. 3, right). This greatly improves the signal to noise ratio of particle images, when compared to recordings using Mie-scattered light from non-fluorescent particles in our previous work.^9^ This allowed for better measurements of displacements and strains within the phantoms.

The recorded particle images were processed using a freely available particle image velocimetry cross correlation algorithm (PIVlab^23^) and particle paths were spatially integrated over time.^17^ The image correlation of subsequent frames was performed in two passes, with subsets of 64 and 32 pixels and with an overlap of 50%. This resulted in a spatial resolution of 0.33 mm. The two-dimensional displacements were smoothed by linear local regression, with a subset size of 10 × 10 material points, prior to the strain computation in order to reduce noise in the strain tensors.^21^ Spatially smoothing displacements in this way, instead of a previously used median filter,^17^ resulted in reduced noise from the spatially highly-resolved gradient-dependent strains. This led to more continuous and consistent strain contours of the measurements across experiments. Green-Lagrangian strains were computed by forming bilinear finite elements such that the element nodes coincide with the subset centroids of the correlation grid, using a Matlab-based digital image correlation toolbox.^8^ Strains were computed using the displacement gradients derived from the finite elements. From the axial, radial and shear strains, the effective strain *ɛ*_eff_ was computed as:

1 $$\varepsilon_{\text{eff}} = \sqrt {\frac{2}{3} \left( {\varepsilon_{1}^{2} + \varepsilon_{2}^{2} } \right)} .$$

Here, *ɛ*_1,2_ are the maximum and minimum principal strains. *ɛ*_eff_ was used to evaluate the resulting state of strain around the needle, a measurement that has been previously associated to damage in tissue.^1^ For all displacements and strain results, data in the area delimited by a 3 mm radius in the neighborhood of the needle was considered.

## Results

### Insertion Force Profiles

As internal optical measurements could only be obtained for the transparent phantoms, only MCH and gelatin were analyzed in this way, allowing the observed differences in forces to be correlated with measured internal displacements and strains.

**Figure 5:**
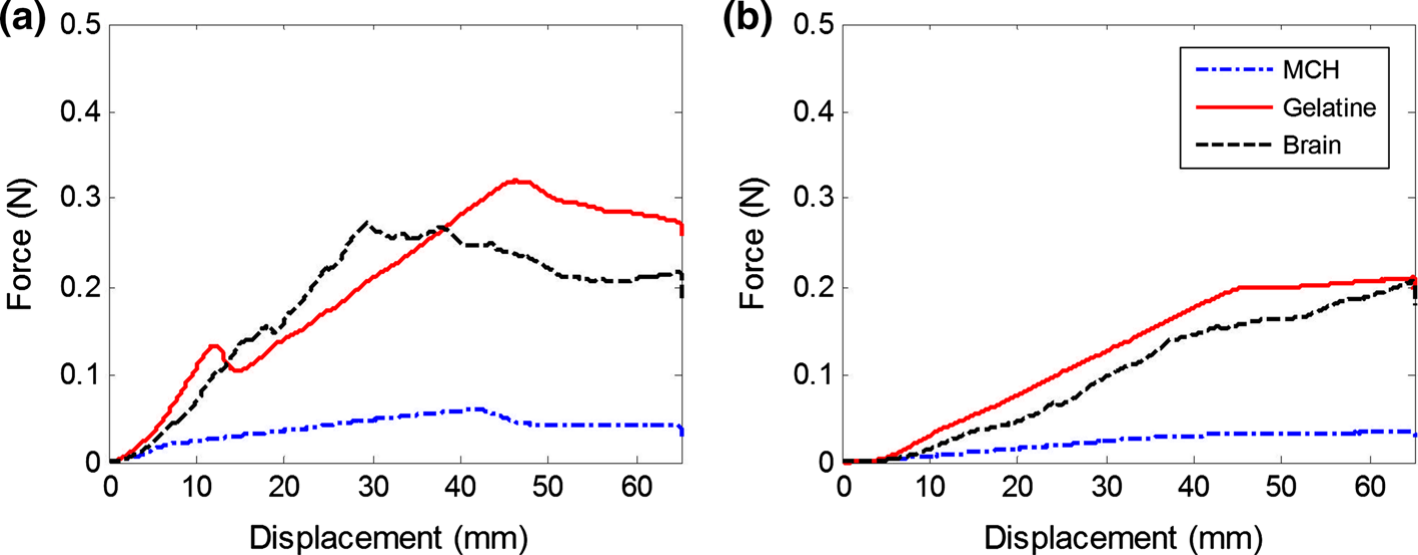
Mean forces of the needle for insertions (a) and reinsertions (b) into MCH, gelatin and porcine brain. Left are the forces of the first insertions and right are the reinsertions into the existing crack, which were performed directly after the first one.

The rigid needle was inserted at a speed of 1 mm/s into MCH, gelatin and porcine brain. All samples were contained in identically sized boxes. The mean insertion forces for the first and the second insertion into the existing crack are shown in Fig. 5. In gelatin, the first peak and the following drop corresponds to the instant of puncture and subsequent relaxation of the substrate. Whereas puncture for the gelatin samples always occurred at the surface, punctures in brain took place at varying depths, likely due to its heterogeneous inner structure. Therefore, unlike for gelatin, the mean force profile in the brain does not show a clear puncture, and is less smooth in general throughout the insertion profile. For MCH, no peak in force prior to cutting was observable, implying immediate puncture by the needle.

After puncture, forces increase steadily with insertion depth to overcome friction between the progressing needle and the inner crack surface. After exiting the sample, towards the end of the insertion (Fig. 5a), there is no more cutting resistance and forces drop and become more stable in overcoming only the frictional resistance (sliding force). Gelatin shows both the highest peak force and sliding force. The increasing slope during cutting is similar between brain and gelatin, but the force after exiting the sample for brain is below that of gelatin. MCH shows a much lower cutting slope and sliding force compared to both brain and gelatin. Overall, the insertion force profiles of brain and gelatin are well matched and closer than those of brain and MCH.

Expectedly, the reinsertion forces (Fig. 5b) are generally lower than for the first insertions, because the needle does not need to cut as it travels through the pre-formed crack. This makes the force profiles insensitive to cutting-related variations, such as those caused by inner membranes in the brain, and reduces the spread between trials. Measured forces for gelatin and brain agree even more closely than for first insertions in both slope and sliding force, although the forces for gelatin are slightly higher throughout the reinsertion. As before, the profile for MCH is much lower overall and the difference between first insertion and reinsertion is smaller than for the other two materials.

### Interactions During Needle Cutting

**Figure 6:**
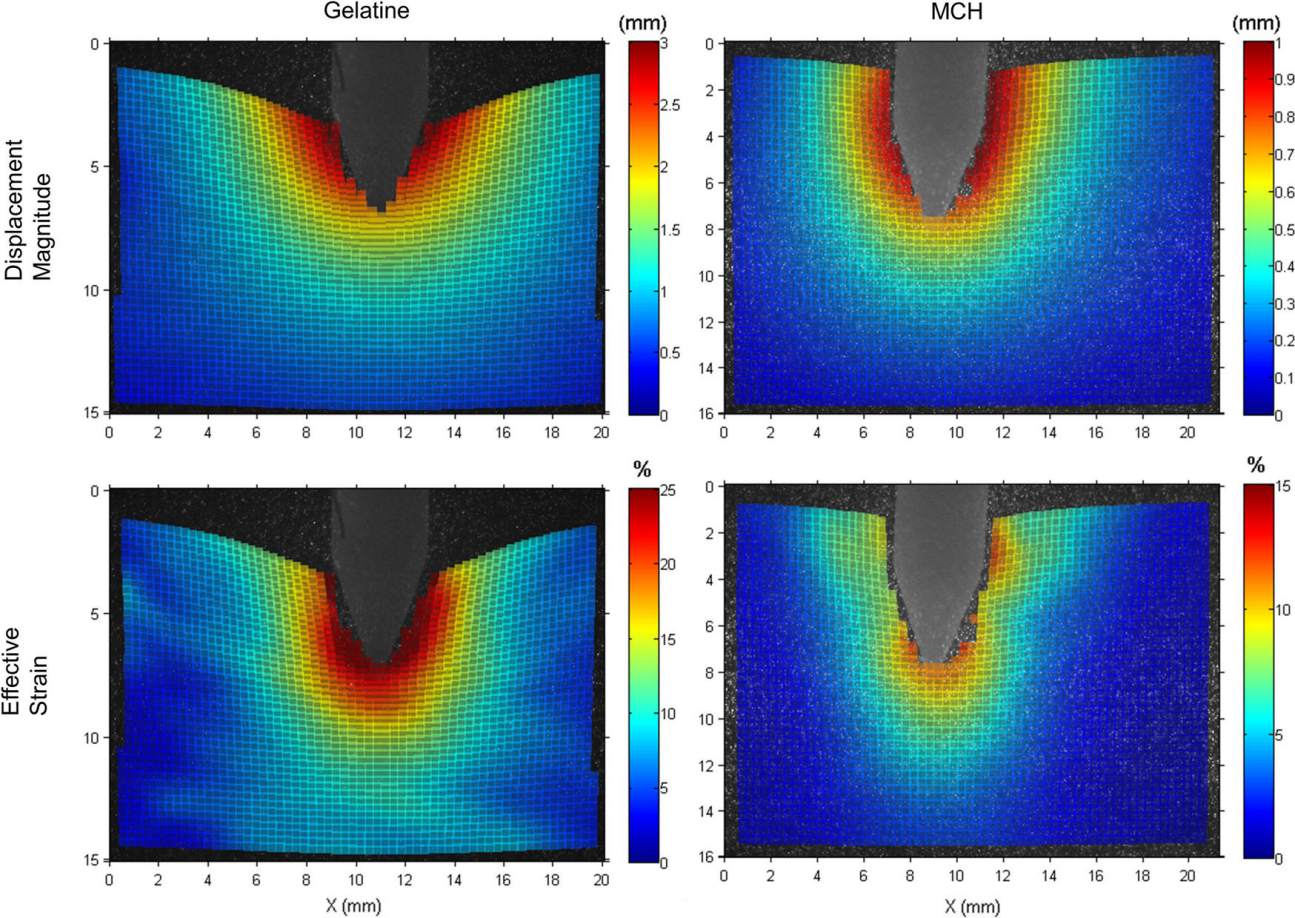
Contour plots of the imaging results during a needle insertion for both phantom materials, gelatin (left) and MCH (right). The first row shows displacement magnitude calculated from axial and radial displacements. The second row shows the effective strain obtained from the Green-Lagrangian strains (see Eq. 1).

Additionally to the measured insertion forces, optical measurements of displacements and strains within the transparent phantoms provides a comprehensive understanding of interactions between the needle and the substrates. Figure 6 shows the displacement magnitude and effective strain that develop around the needle, in both gelatin and MCH, during cutting, at an insertion depth of 20 ± 2 mm. Displacements and strains become higher towards the needle, as the surrounding material is deformed during crack formation. Strains in front of the needle are highest, as this is the area where material is closest to failure. Figure 6 also illustrates how, in gelatin, the top interface of the material is dragged further in the direction of the needle motion, resulting in the overall displacements and strains being distinctly higher than for MCH. Regions further away from the needle surface are more affected in gelatin. Whereas displacements in MCH become negligible (smaller than 0.5 mm) at a distance of approximately 3 mm from to the needle surface, the same region in gelatin shows much higher values, of around 1.5 mm. Strain in MCH is also more concentrated, with local peaks ahead of the needle and at the tip-to-shaft transition, but with strain values of only about 5% on the bevel tip edges. Conversely, strains in gelatin are above 25% around the entire needle tip.

**Figure 7:**
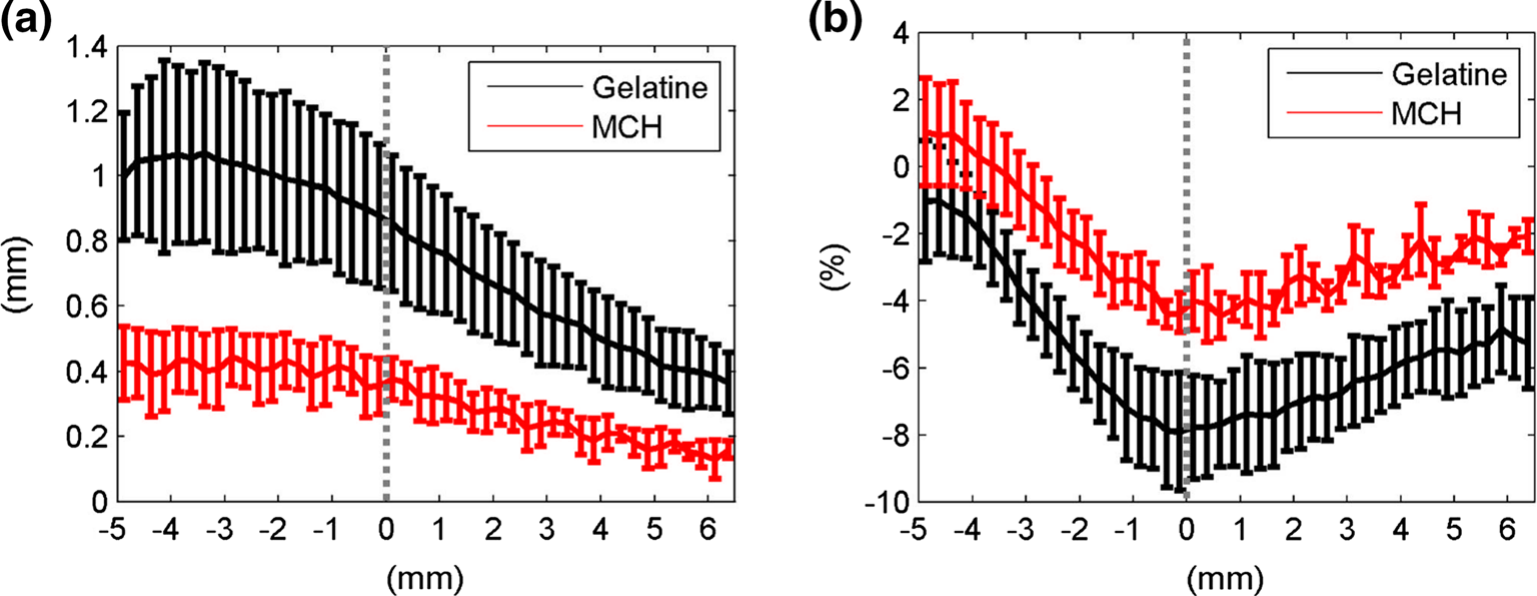
Mean axial displacements (a) and strains (b) in the needle surroundings for all experiments, with standard deviations, along the needle axis whilst cutting. The *x*-coordinate represents the position along the needle axis and the needle tip is marked at *x* = 0.

For a more detailed analysis of interactions during cutting, Fig. 7 shows the mean axial displacements and strains along the needle axis during cutting, at a depth of 20 ± 2 mm. The needle tip is indicated at *x* = 0, with negative *x*-values being positions along the shaft and positive ones being ahead of the needle. This figure shows that the material is dragged along and pushed ahead of the needle, leading to a peak in compressive strain in front of the needle. Displacements and strains are significantly higher in gelatin along the needle shaft and ahead of the tip.

### Displacements and Strains in Needle Surroundings

The large difference in displacement and strain patterns observed near the needle is also seen in the material surrounding the needle shaft, when it is at its deepest point after puncturing and travelling through the sample for an additional 20 mm. Table 1 shows the measured mean displacements for gelatin and MCH axially and radially to the needle, and the effective strain. For gelatin, the axial displacement is nearly four times higher than MCH, indicating that the material is dragged further with the needle. Radial displacements are almost half of the axial displacements. Conversely, MCH shows a reversed pattern of displacements, with significantly higher radial displacements than axial ones. The effective strain in the needle surroundings during sliding is lower for MCH than for gelatin.

**Table 1:** Averaged final displacements and strains in both phantoms around the needle, at the deepest location after through puncture.

	Axial displacement (mm)	Radial displacement (mm)	Effective strain (%)
Gelatin	1.76 (0.45)	0.87 (0.30)	14.31 (3.04)
MCH	0.48 (0.22)	1.14 (0.10)	10.42 (0.65)

### Transient Response

**Figure 8:**
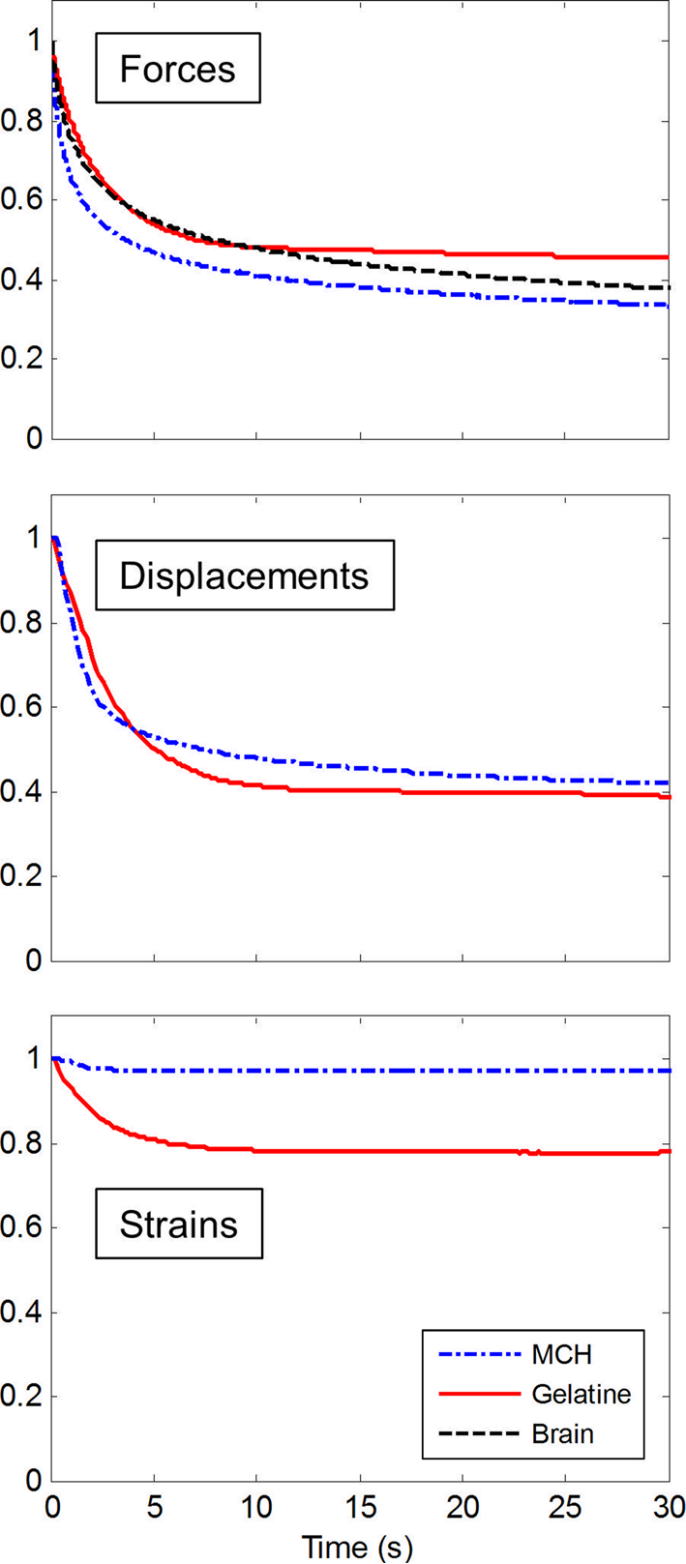
Means of the normalized time responses whilst the needle was held in position after travelling through the samples. For insertions into porcine brain, only force data exists.

During the experiments, the needle was held in place for 30 s, after having travelled through the samples for 20 mm. Figure 8 shows the averaged transient response of brain, MCH and gelatin relative to the initial state, during the period for which the needle remained stationary. Measured forces and responses of the mean axial displacements and effective strains in the needle surroundings are shown, with displacement and strain data only being available for the two transparent phantom materials. The approximate slope at 10 and 30 s was computed for forces, displacements and strains (Table 2).

**Table 2:** Values for the slopes of the transient responses between 10 and 30 s during which the needle was held stationary.

Transient slope (×10^−2^ s^−1^)	MCH	Gelatin	Brain
Force	−0.392	−0.116	−0.516
Displacement	−0.303	−0.130	n/a
Strain	0.003	−0.011	n/a

Forces for brain and gelatin initially drop at similar rates, but, whereas gelatin forces reach a stable level around 50% after approximately 10 s, the forces for brain continue to decrease throughout the 30 s duration. While for MCH the initial drop of forces is more pronounced than that for brain and gelatin, MCH shows an on-going decrease and a negative slope after 10 s, which is 75% of the slope observed for brain. Gelatin shows a slope of only 23% compared to brain for the same period.

The different transient responses between MCH and gelatin are also observed in the surrounding displacements of the needle, which are still decreasing at more than twice the rate for MCH than for gelatin. In contrast to forces and displacements, for MCH, strains remain fairly stable during the entire period. Strains in gelatin drop initially by less than 20% and then remain virtually unchanged.

## Discussion

Gelatin and composite hydrogels, which are used to mimic soft tissue, were stiffness-matched to porcine brain. The material behaviors that correspond to tool-tissue interactions of the two transparent phantoms, gelatin and MCH, were analyzed during needle insertions. A laser-based image correlation technique allowed measuring internal displacements and strains at high resolution. The use of fluorescent particles improved the imaging contrast and led to optical measurements of internal displacements and strains with reduced noise compared to previous work, using non-fluorescent particles. Insertion forces, displacements, and strains built a comprehensive set of data that could be used to assess the phantom materials’ behavior compared to real brain for fracture, friction and time-dependency.

The transparency of gelatin and MCH makes the materials suitable for the detailed investigation of tool-tissue interactions close to the needle interface with high resolution, which would not be possible with typical material testing systems. This has allowed us to better understand and model the complex mechanisms during needle insertions that arise from frictional interaction, large deformations and cutting. The results demonstrate how differently phantom materials behave when tool-tissue interactions are investigated. Since the elastic responses of gelatin and MCH were initially matched to porcine brain, the observed differences in forces, displacements and strains between materials must stem from tool-tissue interactions, crack formation, and the inner friction between the needle and the developing crack.

The measured insertion forces are dependent on fracture and frictional properties. The difference between forces required for insertion and reinsertion highlights the contribution of cutting to the insertion force profile. The close match between brain and gelatin for insertion and reinsertion indicate similar inner friction between needle shaft and the material and a similar resistance to cutting. Despite the observation that both materials have contrasting fracture behavior, the force required for the needle to cut proves to be similar due to similar fracture toughness. The forces observed for MCH are overall significantly lower. At insertion, the low fracture toughness causes low resistance and immediate puncture. The significantly reduced frictional resistance, as can be observed after through puncture, is partly due to the separate phase network of MCH; PVA dissolved as a filler phase produces a thin wet film between the needle and the material.

The higher friction of gelatin causes the material to be dragged further, with the needle and its higher toughness causing significantly higher strains at the needle tip than in MCH (Fig. 7). Due to such interactions, regions further into the substrate and away from the needle are affected and boundary conditions become more important.

Thus, with the same needle, and with the elastic behavior of phantoms matched at small strains, final configurations around the needle can be divergent at the end of the insertion. This is demonstrated by the higher axial displacements in gelatin due to higher friction, but lower radial displacements than for MCH. The higher level of effective strain in gelatin is primarily caused by the axial motion of the material towards the bottom wall of the sample box, leading to compressive axial strains.

Although gelatin matches brain well for interactions due to friction and fracture, it does not capture transient responses of soft tissue due to its low viscous nature. These results agree with the material characterization discussed in “Materials” section. In comparison, MCH, with its composite structure, matches the decrease in force of brain over time better because of its close agreement in viscous properties to brain (Fig. 1b). The same relationship between the two phantoms can be observed in the displacements (Fig. 8), which show a continuous drop in displacements around the stationary needle when the substrate is viscous. Over time, strains in the MCH around the needle remain stable. This indicates little deformation of the substrate along the shaft, as soon as the needle comes to a stop.

Frictional properties of brain were best matched by gelatin. However, gelatin showed a more brittle fracture behavior than brain, which could lead to different strains distributions close to the needle, than within soft tissue. This would need to be taken into account when selecting a phantom for investigations where these phenomena are important, e.g., flexible needles used to steer within tissue, since these rely on strains at their tip to flex and cut.

The transient response of brain in the needle surroundings is best matched by MCH. Since medical interventions involve some form of delivery (e.g., deep brain stimulation, brachytherapy) or removal (e.g., biopsy), the changing state around a stationary needle may be pertinent. This may be of particular importance if the procedure relies on pre-operative imaging or if the frequency of image acquisition is low. Thus, for studies that investigate medical procedures involving time-dependent interactions, consideration must be made as to whether viscous effects are negligible or not. The modified preparation procedure of MCH showed much lower stresses and strains at the point of failure than for CH, leading to low resistance to cutting by the needle.

All of these findings are succinctly summarized in Table 3, which provides a straightforward guide to material selection, given the intended application, required phantom characteristics, and experimental scenario. Indeed, while none of the materials analyzed match the behavior of biological soft tissue in full, each type presents valuable attributes (stiffness, viscosity, transparency, *etc*.) which, if suitably paired to a given problem, should provide the degree of fidelity required to reduce our reliance on *ex vivo* testing.

**Table 3:** Summary table for the comparison of the investigated phantom materials with brain tissue.

Matching criterion	Gelatin	CH	MCH
Stiffness	✓	✓	✓
Relaxation		✓	✓
Inner friction	✓	n/a	
Fracture resistance		✓	
Transparency	Yes	No	Yes
Example application	Quasi-static scenarios e.g., surgical needle steering	Highest fidelity e.g., brain shift simulation^4^	Dynamic scenarios e.g., tool-tissue interaction experiments

The tick mark indicates the satisfactory mimicking of the respective material behavior of real tissue

## Conclusions and Outlook

Gelatin and composite hydrogels are often used in a variety of experiments, which aim to assess the performance of instruments within biological soft tissue. However, works in the literature capture a wide spread in the results based on these synthetic media, likely because of the necessity to tune their performance to match tissue characteristics. In this work, we have strived to evaluate the performance of composite hydrogels and gelatin against a number of material properties, with the aim to provide the reader with a key to the selection process, based on their intended experimental setup and end goal.

The investigated materials were shown to perform differently in terms of several material parameters that influence tool-tissue interactions. Gelatin, which is the most commonly used phantom material in the literature, showed similar behavior to brain during cutting, but low viscoelasticity. The composition of CH can potentially be tuned to match any of the tissue characteristics, but is opaque and thus of limited value in experimental scenarios where line of site is important. MCH, with its transparency and better viscous properties compared to gelatin, can be used for detailed interaction studies, but showed low cutting resistance.

In summary, gelatin and composite hydrogels match different characteristics of brain during needle insertions, but neither is a perfect substitute for mimicking its complete behavior. Thus, the selection of phantom materials should be informed by both the special material properties of the soft tissue reference and the complex needle-tissue interactions needing to be replicated. This ensures that findings generated with these substitute materials better reflect real life conditions. New developments, such as those in the areas of tool design or needle control, must be extensively investigated using methodologies that are independent of tissue-specific variations. A better understanding of brain-mimicking phantoms can potentially accelerate this process.
